# Abstracts from the 22nd International Symposium on Signal Transduction at the Blood–Brain Barriers

**DOI:** 10.1186/s12987-019-0149-2

**Published:** 2019-09-11

## A1 The brain barriers: gatekeepers of CNS immune privilege

### Britta Engelhardt

#### University of Bern, Theodor Kocher Institute, Bern, Switzerland

##### **Correspondence:** Britta Engelhardt - bengel@tki.unibe.ch

*Fluids Barriers CNS* 2019, **16(2):** A1

Discoveries leading to an improved understanding of immune surveillance of the central nervous system (CNS) have repeatedly provoked dismissal of the existence of immune privilege of the CNS. Recent rediscoveries of lymphatic vessels within the dura mater surrounding the brain by modern live cell imaging technologies have revived this discussion. Understanding immune privilege of the CNS requires intimate knowledge of its unique anatomy. Employing in vivo and in vitro studies on immune cell migration into the CNS during immune surveillance and neuroinflammation using live cell imaging we have explored the anatomical routes and cellular and molecular mechanisms involved in immune cell trafficking to the CNS. Our findings underscore that endothelial, epithelial and glial brain barriers establish compartments within the CNS that differ strikingly with regard to their accessibility to immune cell subsets.

**Grant support:** This work was supported by the Swiss National Science Foundation, Fidelity International Foundation.

## A2 Contribution of the blood–brain barrier in Alzheimer’s disease, mouse modelling

### Amaia Dominguez-Belloso^1^, Ricardo Figueiredo^2^, Sylvaine Guérit^1^, Sonja Thom^1^, Kavi Devraj^1^, Peter Winter^2^, Stefan Liebner^1^

#### ^1^Institute of Neurology (Edinger-Institute), Johann Wolfgang Goethe-University Frankfurt, Medical School, Heinrich-Hoffmann-Straße 7, 60528, Frankfurt, Germany; ^2^GenXPro GmbH, Altenhöferallee 3, 60438, Frankfurt, Germany

##### **Correspondence:** Amaia.dominguez-belloso@kgu.de

*Fluids Barriers CNS* 2019, **16(2):** A2

The blood–brain barrier (BBB) protects the brain from the detrimental influence of various blood-born, endogenous and exogenous substances, and is known to be directly or indirectly involved in the progression of brain pathologies. Although growing evidence points to a major role of the brain vasculature and the BBB in disease progression, little is known about the specific processes taking place at the neurovascular unit (NVU). In order to shed light on the events taking place at the NVU in brain diseases, we performed next generation sequencing, employing the MACE technology, of mouse brain microvessels (MBMVs) from an Alzheimer’s disease (AD) mouse model, harbouring the Thy1-SwDI triple mutation of amyloid precursor protein (APP), and compared it to aged-matched WT mice at 6 and 18 month-of-age. We show that particularly at 6 month-of-age significant regulation of genes involved in cellular junctions, transporters and angiogenic processes takes place in AD mice. Along with these changes in the expression profile we observed that AD mice have memory and BBB deficits at 6 month-of-age by behavioural and tracer experiments with 3-4KD dextrans, respectively, which became aggravated with age. Interestingly, differences in gene expression between AD and WT mice were less pronounced at 18 month-of-age, possibly suggesting that ageing per se leads to pronounced changes at the NVU, and that the AD condition might only push the system to become pathologic. Together, our results point into a major contribution of a hampered BBB in AD but also in ageing, reemphasizing the vascular aspect of AD and highlighting the BBB as a possible therapeutic target.

## A3 3Rs in blood–brain barrier research: The power of in vitro models

### Winfried Neuhaus

#### AIT Austrian Institute of Technology GmbH, Vienna, Austria

##### **Correspondence:** Winfried Neuhaus - winfried.neuhaus@ait.ac.at

*Fluids Barriers CNS* 2019, **16(2):** A3

The principles of the 3Rs concept to alternatives to animal testing were first introduced in 1959 by Bill Russel and Rex Burch in their groundbreaking book “The Principles of Humane Experimental Technique”. Basically, it deals with measures to refine, reduce or replace animal experiments with completely animal-free methods. Although the 3Rs are often stated in research proposals as a goal for method development, success in the sense of the 3Rs is very limited. Hardly any method is developed so far that it can really replace an animal experiment specified by the regulations. In the cosmetics industry, animal experiments have been banned in the EU, in toxicology there is a clear trend towards alternative methods, and in the pharmaceutical industry a rethinking of the sensible use of animal experiments is slowly beginning. Nevertheless, the number of animal experiments is increasing, mainly due to the increased use in basic science. It is important to point out that the 3Rs do not serve a purely ethical purpose. With the help of advanced methods, one can pragmatically obtain much better scientific data, especially if one thinks of species differences and considers the translation of data from animal models to humans. In addition, new non-animal methods are cheaper than animal models and can therefore contribute to saving money. In this lecture, an overview of data and new in vitro models in the blood–brain barrier area will be given, which can contribute to the 3Rs, but also their applicability and limitations will be critically examined. In summary, these non-animal models can be used as great, innovative methods to supplement limitations of animal testing and should not be seen as merely replacing and alternatives to animal experiments. In the future, the common, meaningful use of non-animal models with animal experiments could lead to a significant, scientifically meaningful reduction of animal experiments in order to generate even more relevant data for humans.

## A4 Epigenetic regulation of BBB integrity in stroke

### Anuska V. Andjelkovic, Svetlana M. Stamatovic, Chelsea M. Phillips, Gabriela Martinez-Revollar, Richard F. Keep

#### Department of Pathology and Neurosurgery, University of Michigan, Medical School, Ann Arbor, USA

##### **Correspondence:** anuskaa@med.umich.edu

*Fluids Barriers CNS* 2019, **16(2):** A4

Acute cerebral ischemic injury evokes complex cascade of pathophysiological events at the blood–vascular–parenchymal interface, which evolve over time and space, and result in damage to neuronal cells and edema formation. Stroke survivors usually recover at least some functionality within 3 months, although only 25% return to pre-stroke levels. One problem regarding recovery after stroke is that there are ongoing processes during the chronic phase that increase the risk of recurrent stroke and could also be a solid basis for developing post-stroke cognitive impairment (vascular dementia). The new emerging evidence suggest that Blood brain barrier (BBB) recovery is incomplete process which could influent the stroke injury recover, increase risk for the new stroke occurrence and be solid substrata for developing the vascular dementia. Although that several processes are indicated to guide the BBB partial recovery, there are still lack of knowledge whether and how these processes limited the BBB recovery. In the present study we have focused on elucidate the involvement and role of epigenetic mechanism in regulation of post-stroke BBB recovery. The young (3 months) and old mice (18 months) were subject to thromboembolic (TE) stroke based on injection of suspension of platelet-reach microemboli. TE stroke was confirmed and its resolution was followed for 10 days by MRI T2 imaging while BBB permeability was observed and analyzed 1–10 days after stroke using GD-DTPA tracer by MRI T1 imaging. The brain microvessels were collected at day 1 and 10 after TE stroke from area of stroke injury and GD-DTPA hyperpermeability. The isolated brain microvessels from young and old mice were subject of DNA methylation analysis (global DNA methylation assay and Reduced Representation Bisulfite Sequencing (RRBS)) as well histone modification analysis (MODfied Histone peptide Area and ChIP-Seq). Our results indicated profound alteration in DNA methylation and histone monomethylation particularly H3K9, H3K27 and trimethylation (H3K9, H3K79, H3K27) in old mice 10 days after TE stroke. The most affected are proinflammatory and junctional regulator genes. This study provides new information related to the epigenetic regulation of post-stroke BBB and revealed the potential novel therapeutic targets to treat BBB hyperpermeability.

## A5 Restoring Blood–Brain Barrier Function to Improve Cognition in Alzheimer’s Disease

### Anika M.S. Hartz^1,2^, Atcharaporn Ontawong^1,3,4^, Yujie Ding^1^, Chutima Srimaroeng^3^, Bjoern Bauer^5^

#### ^1^Sanders-Brown Center on Aging, University of Kentucky, Lexington, USA; ^2^Department of Pharmacology and Nutritional Sciences, College of Medicine, University of Kentucky, Lexington, USA; ^3^Department of Physiology, Faculty of Medicine, Chiang Mai University, Chiang Mai, Thailand; ^4^Division of Physiology, School of Medical Sciences, University of Phayao, Phayao, Thailand; ^5^Department of Pharmaceutical Sciences, College of Pharmacy, University of Kentucky, Lexington, USA

##### **Correspondence:** Anika MS Hartz - anika.hartz@uky.edu

*Fluids Barriers CNS* 2019, **16(2):** A5

**Introduction:** Increasing evidence indicates that blood–brain barrier dysfunction contributes to cognitive decline in Alzheimer’s disease (AD). Two key elements of barrier dysfunction include (1) reduced levels of the blood–brain barrier transporter P-glycoprotein (P-gp) that clears Aβ from the brain, and (2) barrier leakage. Both reduced P-gp and barrier leakage have been linked to Aβ brain accumulation and cognitive decline. While increasing evidence shows Aβ involvement in barrier dysfunction, the underlying mechanisms remain to be fully defined. Moreover, therapeutic strategies to restore barrier function are currently not available. Thus, there is an unmet critical need to define the mechanism(s) that lead/s to barrier dysfunction and to develop effective intervention strategies to help restore barrier function in AD. In the present study, we test the hypothesis that restoring P-gp through PXR activation and reducing barrier leakage by scavenging reactive oxygen species (ROS) will restore barrier function, increase Aβ clearance, and slow cognitive decline.

**Methods:** To test our hypothesis, transgenic hAPP mice received a purified diet containing the PXR activator pregnenolone 16α-carbonitrile (PCN) or the ROS scavenger *N*-acetyl-l-cysteine (NAC). We assessed P-gp protein expression and transport activity, Aβ brain and plasma levels, capillary leakage, renal function and cognition to test the PCN or NAC effect in hAPP mice.

**Results:** Feeding hAPP mice for 21 months with the PXR activator PCN restored P-gp protein expression and transport activity, lowered Aβ brain levels, and improved cognition. Further, treating 12-month old mice with NAC for 3 weeks improved renal function and reduced barrier leakage.

**Conclusion:** Our findings suggest that a combination therapy of the PXR activator PCN and the reactive oxygen scavenger NAC will restore P-gp levels and reduce the extent of barrier leakage which has the potential to lower brain Aβ burden and slow cognitive decline.

**Grant support:** This work was supported by the 2R01AG039621 (NIA/NIH; PI: Hartz); 1R01AG054459 (NIA/NIH; Co-I: Hartz); P30AG028383 (NIA/NIH; PI: Hartz) grants.

## A6 Polyphenols and their metabolites beyond brain barriers: studies on a sheep model

### Janina Skipor, Malgorzata Domzalska, Aleksandra Szczepkowska, Dorota Szawara-Nowak, Natalia Platosz, Wieslaw Wiczkowski

#### Institute of Animal Reproduction and Food Research, Polish Academy of Sciences, Olsztyn, Poland

##### **Correspondence:** Janina Skipor - j.skipor@pan.olsztyn.pl

*Fluids Barriers CNS* 2019, **16(2):** A6

Natural polyphenols of flavonoid-type (flavonols, flavones, flavanols, flavanones, anthocyanins and isoflavones) and non-flavonoid type (phenolic acids, lignans, stilbenoids) with their anti-oxidative and anti-inflammatory properties are considered as promising feed additives in the nutrition of farm animals. These are present in plants either in their glycosylated (with sugar attached), free aglycones (no sugar) or in the less extended esterified form, which dictates their passage, absorption, and bioavailability in the body. Moreover, the biotransformation of polyphenols in enterocytes and liver to conjugated metabolites (glucuronided, methylated, sulphated and combined) seems to reduce their biological activity. It should be emphasized that, the position of conjugation in phytochemical molecules determines the extent of their actual bioactivities. The studies indicated that some forms of polyphenol metabolites, such as 7-glucuronide and 3-glucuronide exhibited high efficiency as antioxidants. We have been using mature female sheep to study isoflavones (daidzein, genistein), flavonols (quercetin derivatives), anthocyanins (cyanidin derivatives) and their metabolites access to the brain. The sheep model has serious advantages: (1) allows for long-lasting serial sampling of peripheral blood parallel with cerebrospinal fluid from the cerebral ventricles, (2) can be done with freely moving animals and, thus, avoids many of the potential complications associated with restraint, (3) the use of ovariectomised, estradiol implanted ewes preserves estradiol receptors in the brain and prevents the variability resulting from the changing level of sex hormones.

**Grant support:** This work was supported by KNOW (Leading National Research Centre) Scientific Consortium “Healthy Animal - Safe Food”, decision of Ministry of Science and Higher Education No. 05-1/KNOW2/2015, and by the National Science.

## A7 Mechanisms and Regulation of Brain Iron Uptake

### James Connor, Ian Simpson

#### Penn State University, State College, USA

##### **Correspondence:** James Connor - jconnor@pennstatehealth.psu.edu

*Fluids Barriers CNS* 2019, **16(2):** A7

Iron delivery to the brain is essential for multiple neurological processes such as myelination, neurotransmitter synthesis and, as it is for all organs, for the utilization of oxygen for energy production. Previously, we introduced and established the concept that brain iron uptake is regulated at the level of the endothelial cells of the blood–brain barrier (BBB), which is contrary to the widely held concept that the BBB endothelial cells serve as a simple conduit for the delivery of transferrin. We also identified H-ferritin, classically considered an intracellular iron storage protein, as a significant iron delivery protein for the brain. These new concepts start to address the question of how the brain acquires iron in a timely manner and adequate amounts. The importance of timely iron delivery during development is clinically manifested in the long term neurological and cognitive impact of developmental iron deficiency. The importance of regulation to adequately manage the amounts of iron delivered in the adult brain is clinically manifested in neurological disorders such as Restless Legs Syndrome (too little iron) and neurodegenerative diseases (too much iron). Despite the prevailing opinion that the BBB was a simple conduit with no apparent mechanism for regulation of brain iron uptake, we identified age, genotype, sex, and systemic iron status as physiological factors that modulate brain iron acquisition. Moreover, we have identified H-ferritin (FtH1) as a significant brain iron delivery protein, particularly during development that augments the traditional transferrin delivery system. Both proteins deliver iron to the brain during development in a mouse model but the pattern for the two proteins is significantly different. We also examined the impact of a mutant form of the human iron homeostatic regulator protein (HFE) protein, which is the most common gene variant in Caucasians. We demonstrate that at postnatal day 22, mutant mice brains take up greater amounts of iron compared to wildtype. In summary, brain iron uptake is mediated by two distinct mechanisms both of which are modulated by age, sex and genotype.

**Grant support:** This work was supported by NIH 5R01NS077678.

## A8 Pericyte nanotubes and microtubes: Are they both related to brain microvessel growth?

### Ignazio De Trizio^1, 2^, Mariella Errede^2^, Francesco Girolamo^2^, Giovanna Longo^2^, Roberto Perris^3^, Daniela Virgintino^2^

#### ^1^Department of Neurosurgery, Neurocenter of Southern Switzerland, Regional Hospital Lugano, Lugano, Switzerland; ^2^Department of Basic Medical Sciences, Neurosciences and Sensory Organs, Bari University Medical School, Bari, Italy; ^3^COMT-Centre for Molecular and Translational Oncology & Department of Chemical and Life Sciences and Environmental Sustainability, University of Parma, Parma, Italy

##### **Correspondence:** Ignazio De Trizio **-** ignazio.detrizio@gmail.com

*Fluids Barriers CNS* 2019, **16(2):** A8

**Introduction:** In 1897, a woman, Julia Platt, established that neural crest cells (NCCs) in the head participate in the formation of high variety of mesenchymal derived tissues and created the term mesectoderm (now ectomesenchyme) for the mesenchyme of neuroectodermal origin able to develop into a large variety of cells, including forebrain pericytes (FPs). In our hypothesis, FPs can act as multitasking neural crest-derived cells and display a unique angiogenic attitude compared to pericytes of a mesodermal origin homing other different vascular district. Pioneering studies of pericyte-endothelial relationships during human foetal brain vascularization, revealed an intimate interplay of these cells in vessel sprouting events, redefining the view of a late recruitment of pericytes and suggesting that FPs can actually lead the nascent vessel growth and anastomosis. Moreover, recent studies have supported the specific angiogenic activities of FPs, showing tunnelling nanotubes (TNTs) in vessel-to-vessel communication during the earliest events of normal as well as brain tumor angiogenic sprouting.

**Methods:** We analysed aspects of FPs embryonic origin and mesenchymal nature, and TNTs and cord-like microtubes (MTs) formation, as possible steps in FP angiogenic activity during both human brain and glioblastoma vascularization, revealing the expression of pericyte, mesenchymal, and neural crest cell markers including NG2, CD146, and Sox10, by high resolution confocal microscopy.

**Results and conclusions:** The results are in agreement with data obtained in murine brain tumor models4 and indicate that during angiogenesis, FPs seem to maintain their original NCC-derived pluripotency, forming TNTs and MTs initially composed solely of pericytes. The leading role of FPs in the earliest events of vascular growth suggests anti-angiogenic therapeutic strategies that combine pericyte TNT and MT targeting.

## A9 BBBHub: an expression datahub for brain barriers

### David Francisco^1^, Rémy Bruggmann^1^, Michal Okoniewski^2^

#### ^1^Interfaculty Bioinformatics Unit, University of Bern, Bern, Switzerland; ^2^ID Scientific IT Services, ETH Zurich, Zurich, Switzerland

##### **Correspondence:** David Francisco - david.francisco@bioinformatics.unibe.ch

*Fluids Barriers CNS* 2019, **16(2):** A9

**Introduction:** Due to the highly specialized nature of the brain barriers tissues, as well as their development and maintenance process, transcriptome profiling is a vital tool to better understand processes in the blood–brain Barrier (BBB). In particular, RNA-seq techniques have provided the opportunity of more thorough studies in many fields of life sciences. Interestingly, the number of studies taking advantage of this technique to study the brain barriers is still very low when compared to the total number of publications concerning the brain barriers. However, we expect that (i) the number of studies using RNA-seq will increase dramatically and (ii) these methodologies will help to bring the knowledge of the BBB to a new level.

We present “BBBHub”, a web based data hub platform that allows (i) the collection of existing and new data (ii) the integration and organization of omics-data (mainly RNA-seq) (iii) the analysis of the data in a standardized fashion and (iv) the access to the data to researchers in the field.

**Methods:** The BBBHub uses as its backbone the frame work “openBIS”, an open source platform for managing scientific information. Furthermore, we will develop an easy-to-use front-end system to allow all researchers to use the without advanced knowledge in computational biology.

**Results/conclusion:** We set up standardized upstream data analysis workflows to allow comparison across datasets and to enable a flexible downstream analysis workflow. Mixed with the search functions this allows for comparison across genes or across whole datasets. These features will be very helpful to understand the utility and limitations of each dataset. In the coming months, we will be adding other features, such as single-cell RNA-seq and isoforms detection workflows.

**Grant support:** This work was supported by the EU H2020-MSCA-ITN-2015 675619 BtRAIN.

## A10 Establishing a stem cell-based model of the blood–brain barrier to analyze barrier permeation of nanoparticles

### Helen Onyema, Anna Musyanovych, Christian Freese

#### Fraunhofer IMM, Germany

##### **Correspondence:** Helen Onyema - Helen.onyema@imm-extern.fraunhofer.de

*Fluids Barriers CNS* 2019, **16(2):** A10

**Introduction:** Due to the blood–brain barrier (BBB) drug delivery to the brain still represents a challenge. Nanoparticles (NP) have the potential to cross this barrier and to function as carrier systems. However, many in vitro models used for studying these transport mechanisms are not of human origin, lack essential characteristics and may therefore limit the translation of the generated results from the laboratories to the clinic.

**Methods:** NP of 100 nm size in diameter were formulated by a combination of miniemulsion and solvent evaporation and, composed of Poly(lactic acid) (PLLA) and Poly(lactic-co- glycolic acid) (PLGA), stabilized by Tween 80. To study the NP-cell-interaction via flow cytometry and microscopy, the NP had a fluorescent dye encapsulated. The physicochemical characteristics of the NP were determined by transmission electron microscopy. Mass spectroscopy was performed to analyze the protein corona. Barrier-building endothelial cells were differentiated from human induced pluripotent stem cells. By measuring the transendothelial electrical resistance (TEER) the barrier formation was investigated. After NP exposure of 20 h, the toxic potential of all NPs was determined via crystal violet, FVS660 and CCK-8 assays.

**Results:** Having low batch-to-batch deviations, only slight differences of the protein corona occurred among the different polymeric compositions. A model of the BBB consisting of human endothelial cells with a TEER of 2000 Ohm cm^2^ for at least 80 h was established. Being highly internalized by the cells, the NP exposure did not have any toxic effects on the cells. The barrier function was mainly unaffected with only one PLGA-NP leading to a significant decrease of the TEER.

**Conclusion:** An in vitro BBB model based on human cells generating a highly tight barrier was established. The NP exposure study points to a promising potential for BBB permeation of these NP and demonstrates the suitability of the BBB model for in vitro transport studies.

**Grant support:** This work was supported by the Evonik Stiftung.

## A11 Development of an isogenic blood brain barrier spheroid model

### Sanjana Mathew^1^, Alevtina Cubukova ^2^, Marco Metzger ^1, 2^, Antje Appelt-Menzel ^1, 2^, Sabrina Oerter^1^

#### ^1^University Hospital Würzburg, Chair Tissue Engineering and Regenerative Medicine, Röntgenring 11, 97070 Würzburg, Germany; ^2^Fraunhofer Institute for Silicate Research ISC, Translational Center Regenerative Therapies TLC-RT, Röntgenring 11, 97070 Würzburg, Germany

##### **Correspondence:** Sanjana Mathew - sanjana.mathew@uni-wuerzburg.de

*Fluids Barriers CNS* 2019, **16(2):** A11

**Introduction:** A variety of blood–brain barrier (BBB) in vitro models have been developed over the years (Avdeef, Deli, Neuhaus, 2015). In human BBB models, based on primary or immortalized human cells, the desired barrier properties can´t be achieved. The differentiation of human induced pluripotent stem cells (hiPSC) into brain capillary endothelial cells (BCEC) is a promising approach to achieve a human BBB model with in vivo like barrier properties (Lippmann et al., 2012, 2014). Although transwell models are in use because of the simplicity in generation, static models do not reflect the shear stress generated by blood flow in physiological conditions. A major drawback is also the lack of direct cellular interactions, only indirect effect of astrocytes and pericytes on BCECs can be explored in transwell co culture models. It is known that in 2D models, endothelial cells undergo a phenotypic drift, including loss of functionality. Urich et al. and Cho et al. have shown that a 3D BBB spheroid model can be generated spontaneously using immortalized or primary cells (Urich et al., 2017; Cho et al., 2013).

**Methods:** We previously established protocols to generate a 2D transwell BBB model with hiPSC-derived endothelial cells, pericytes and astrocytes (Appelt-Menzel et al., 2017) and explored the possibility of using these hiPSC-derived cell types in developing an isogenic spheroid model of the human BBB and characterized the structure and spheroid formation via histology, immunofluorescence and RNA analysis.

**Results:** The three cell types (astrocytes, pericytes and endothelial cells) can self-assemble and form spheroids where astrocytes form a central core, followed by a layer of pericytes and an outer layer of endothelial cells, reflecting the BBB physiology

**Conclusions:** Our 3D hiPSC-generated BBB spheroids are novel and offer the potential for disease modelling and pharmaceutical studies.

**Grant support:** This work was supported by the Graduate School of Life Sciences, Würzburg, IM2PACT Consortium.

## A12 Development of flow-based hollow-fiber in vitro models of the human blood–brain barrier for long-term studies

### Anna Gerhartl^1^, Maria Kirchsteiger^1^, Alexandra Vladetic^1^, Grace Lin^1^, Nadja Pracser^1^, Antje Appelt-Menzel^2^, Marco Metzger^2^, Winfried Neuhaus^1^

#### ^1^Competence Unit Molecular Diagnostics, AIT Austrian Institute of Technology GmbH, Seibersdorf, Austria; ^2^Department of Tissue Engineering and Regenerative Medicine, University Hospital Würzburg, Würzburg, Germany

##### **Correspondence:** Anna Gerhartl - anna.gerhartl@ait.ac.at

*Fluids Barriers CNS* 2019, **16(2):** A12

**Introduction:** In recent years research on the development of flow-based in vitro blood–brain barrier models has increased progressively. Dynamic hollow-fiber in vitro (DIV-) models offer the possibility to study the blood–brain barrier (BBB) under shear stress. In comparison to microfluidic models, one major advantage is that significant amounts of cell mass and medium can be obtained for subsequent analysis.

**Methods:** The immortalized human brain capillary endothelial cell line hCMEC/D3 or human induced pluripotent stem cell derived brain capillary endothelial cells (hiPS-BCEC) (Appelt-Menzel et al., Stem Cell Reports 2017; Lippmann et al., Scientific Reports, 2014), were cultivated with our established DIV-model for up to six weeks. Cell viability and optimal culture conditions were assessed by glucose and lactate measurements with the BioProfile^®^100Plus, whereby BBB integrity was monitored by TEER measurement and permeability studies at different flow rates with the paracellular marker FITC-Dextran 4000 (FD4). Changes at the mRNA level of tight junction proteins including CLDN1-25, ZO1-3, occludin, tricellulin, JAM 1-3 as well as a series of ABC- and SLC-transporters were analyzed with a “BBB-chip”, an adapted high-throughput qPCR Fluidigm chip, and compared to static transwell models.

**Results:** Protocols for the long-term cultivation of hCMEC/D3 or hiPS-BCEC were successfully established. Adjustments of the medium composition showed a serum-dependency not only for cellular growth rates but also for BBB properties of the hCMEC/D3. Paracellular permeability rates of FD4 were dependent on the applied flow rate. Differences in the gene expression between DIV-models and transwell models indicate a complex regulation of BBB properties induced by flow.

**Conclusion:** The established DIV-models are suitable for long-term studies of the human BBB for six weeks or up to several months offering the possibility of chronic disease-modeling such as Alzheimer’s disease.

**Grant support:** This work was supported by the Stiftung SET zur Förderung der Erforschung von Ersatz- und Ergänzungsmethoden zur Einschränkung von Tierversuchen, project 060 to W.N. and M.M.

## A13 Effects of Tumor Treating Fields (TTFields) on blood brain barrier (BBB) permeability in vivo

### Carsten Hagemann^1^, Almuth F Kessler^1^, Ellaine Salvador^1,2^, Dominik Domröse^1^, Malgorzata Burek^2^, Catherine Tempel Brami^3^, Tali Voloshin Sela^3^, Moshe Giladi^3^, Ralf-Ingo Ernestus^1^, Carola Förster^2^, Mario Löhr^1^

#### ^1^Department of Neurosurgery, Tumorbiology Laboratory, University of Würzburg, Würzburg, Germany; ^2^Department of Anesthesia and Critical Care, University of Würzburg, Würzburg, Germany; ^3^Novocure Ltd, Haifa, Israel

##### **Correspondence:** Carsten Hagemann - hagemann_c@ukw.de

*Fluids Barriers CNS* 2019, **16(2):** A13

**Introduction:** Tumor Treating Fields (TTFields) have been established as adjuvant therapy for glioblastoma patients. The blood brain barrier (BBB) tightly controls the influx of the majority of compounds from blood to brain and may hinder delivery of drugs for treatment of brain tumors. We assessed the influence of TTFields on BBB permeability in vivo.

**Methods:** For 72 h rats were treated with 100 kHz TTFields and thereupon i.v. injected with Evan’s Blue (EB) which directly binds to Albumin. EB was extracted after brain homogenization and quantified to evaluate effects on the BBB. To assess vessel structure, cryosections of rat brains were prepared following TTFields application and stained for tight junction proteins Claudin-5 and Occludin, respectively, and for immunoglobulin G (IgG). In addition, serial dynamic contrast-enhanced DCE-MRI with Gadolinium contrast agent was performed before and after TTFields application.

**Results:** EB accumulation in the rat brain was significantly increased by TTFields application. The vessel structure became diffuse compared to control cryosections of rat brains in treated animals; Claudin 5 and Occludin were delocalized and IgG was found throughout the brain tissue. Serial DCE-MRI demonstrated significantly increased accumulation of Gadolinium in the brain, observed directly after 72 h of TTFields application. The effect of TTFields on the BBB disappeared 96 h after end of treatment and no difference in contrast enhancement between controls and TTFields treated animals was detectable.

**Conclusion:** Application of TTFields at 100 kHz may have the potential to deliver drugs usually unable to cross the BBB to the brain by altering BBB integrity and permeability. Utilizing TTFields to open the BBB and its subsequent recovery could be a clinical approach of drug delivery for treatment of brain tumors and other diseases of the central nervous system.

**Grant support:** This work was supported by Novocure Ltd.

## A14 Cargo-conjugation to Tat affects its potential to facilitate brain endothelial cell adherence, uptake, and barrier permeation

### Mie Kristensen^1^, Eduardo Fernandes^2^, Anders Bach^2^, Kristian Strømgaard^2^, Birger Brodin^1^

#### ^1^Department of Pharmacy, University of Copenhagen, Copenhagen, Denmark; ^2^Department of Drug Design and Pharmacology University of Copenhagen, Copenhagen, Denmark

##### **Correspondence:** Mie Kristensen - mie.kristensen@sund.ku.dk

*Fluids Barriers CNS* 2019, **16(2):** A14

**Introduction and purpose:** The 11-residue cationic peptide Tat1 is a promising tool to facilitate delivery of peptide drug entities not only into cells but likely also across biological barriers, such as the blood–brain barrier (BBB). Conjugation of a cargo to Tat may, however, influence on the permeation properties of the resulting Tat-cargo construct.

In order to evaluate whether Tat conjugation is a viable strategy for brain delivery of drug entities, we evaluate the chemical stability and the BBB interacting and permeating properties of Tat as well as of Tat conjugated to the clinically relevant peptide drug entities NR2B9c (1 kDa)2 and N-dimer (2.1 kDa)3. All constructs were fluorophore-labelled for quantification and visualization purposes.

**Methods and results:** The chemical stability of NR2B9c, Tat, Tat-NR2B9c, and Tat-N-dimer was assessed by UPLC in PBS and cell media supplemented with 10% FBS. All constructs were stable in PBS. In cell media supplemented with 10% FBS ~ 90% of initial NR2B9c and Tat-N-dimer remained intact following 4 h incubation, whereas only ~ 50% and ~ 30% of initial Tat and Tat-NR2B9c, respectively, remained intact.

The propensity of Tat to facilitate brain endothelial cell adherence -and uptake as well as BBB permeation of NR2B9c and the N-dimer was assessed using primary bovine brain endothelial cells cultured in contact with rat astrocytes4. Tat facilitated endothelial cell membrane adherence and uptake of NR2B9c to a greater extent than the N-dimer. In addition, uptake of Tat and Tat-NR2B9c, but not NR2B9c and Tat-N-dimer, was observed in astrocytes cultured on the basolateral (“brain”) side. Following a 3 h BBB permeation study, Tat-NR2B9c appeared to permeate the barrier to a greater extent that NR2B9c, Tat, and Tat-N-dimer.

**Conclusions:** Conjugation of Tat to NR2B9c did not affect cell membrane adherence and cell uptake, whereas conjugation to the N-dimer limited cell membrane adherence and cell uptake. On the other hand, the Tat-N-dimer remained intact in cell media; whereas extensive degradation was observed of Tat and Tat-NR2B9c. Near future studies will elucidate whether the superior BBB permeation of Tat-NR2B9c, when quantified via the conjugated fluorophore, is due to enhanced permeation of a degradation product or reflects permeation of the intact construct.

**Grant support:** This work was supported by the Lundbeck Foundation Research Initiative on Brain Barriers and Drug Delivery (RIBBDD).

**References**

1. Vivès E, et al. A truncated HIV-1 Tat protein basic domain rapidly translocates through the plasma membrane and accumulates in the cell nucleus. J Biol Chem. 1997;272(25):16010–16017.
2. Aarts M, et al. Treatment of ischemic brain damage by perturbing NMDA receptor- PSD-95 protein interactions. Science. 2002;298(5594):846–850.
3. Bach A, et al. Treatment of ischemic brain damage by perturbing NMDA receptor-PSD-95 protein interactions. Proc Natl Acad Sci. 2012;109(9):3317–3322. 10.1073/pnas.1113761109.
4. Helms HC, Brodin B. Generation of primary cultures of bovine brain endothelial cells and setup of cocultures with rat astrocytes. Methods Mol Biol. 2014;1135:205–211.

## A15 Endothelial antigen-presentation stops the migration of CD8+ T cells across the blood–brain barrier in neuroinflammation

### Sidar Aydin, Vivianne Schallenberg, Armelle Klopstein, Britta Engelhardt

#### University of Bern, Theodor Kocher Institute, Bern, Switzerland

##### **Correspondence:** Sidar Aydin - sidar.aydin@tki.unibe.ch

*Fluids Barriers CNS* 2019, **16(2):** A15

**Introduction:** Multiple sclerosis (MS) is an autoimmune disease targeting the central nervous system (CNS). Blood–brain barrier (BBB) breakdown and immune cell infiltration into the CNS are early hallmarks of CNS lesion formation and thus, critically contribute to MS pathogenesis. The high numbers of CD8+ T cells found in MS lesions point to a critical role of CD8+ T cells in MS. The mechanisms mediating CD8+ T-cell migration across the BBB into the CNS are incompletely understood and it has been suggested that endothelial antigen (Ag)-presentation contributes to CD8+ T-cell entry into the CNS.

**Methods and results:** We used primary mouse brain microvascular endothelial cells (pMBMECs) as an in vitro model for BBB. Co-incubation of T-cell receptor transgenic naive OT-I CD8+ T-cells with wild-type (WT) or β2-microglobulin deficient (B2M KO) pMBMECs that were pulsed with the cognate SIINFEKL peptide Ag showed that naive OT-I CD8+ T cells were activated in an Ag- and MHC-Class I dependent fashion. We next investigated, if naive CD8+ T cells can interact with pMBMECs under physiological flow. By employing in vitro live cell imaging, we observed that naive CD8+ T cells were capable to arrest on stimulated pMBMECs and showed decreased motility in the presence of MHC class I and cognate antigen. This prompted us to investigate how endothelial Ag-presentation contributes to the interaction of effector OT-I CD8+ T-cells with pMBMECs under physiological flow. We found that the presence of SIINFEKL and MHC class I on WT pMBMECs prohibited T-cell crawling, decreased the migration rates and allowed for rapid OT-I mediated apoptosis of pMBMECs.

**Conclusion:** Our observations show that MHC-class I mediated Ag-presentation does not facilitate CD8+ T-cell migration across the BBB. This process rather induces a stop signal for CD8+ T cells on the luminal side of the BBB, eventually leading to endothelial cell death and focal BBB breakdown, which is a critical hallmark of MS pathogenesis.

## A16 Developing an hiPSC-derived 3D in vitro model of the blood–brain barrier to study COL4A1/2 cerebral small vessel disease

### Mary Goodwin^1^, Tom Van Agtmael^2^, Karen Horsburgh^3^, Tao Wang^4^, Zameel Cader^5^, Stuart Allan^6^, Sanjay Sinha^7^, Hugh Markus^1^, Alessandra Granata^1^

#### ^1^Stroke Research Group, Department of Clinical Neurosciences, University of Cambridge, Cambridge, UK; ^2^Institute of Cardiovascular & Medical Sciences, University of Glasgow, Glasgow, UK; ^3^Centre for Discovery Brain Sciences, University of Edinburgh, Edinburgh, UK; ^4^Division of Evolution and Genomic Sciences, University of Manchester, Manchester, UK; ^5^Nuffield Department of Clinical Neurosciences, University of Oxford, Oxford, UK; ^6^Division of Neuroscience and Experimental Psychology, University of Manchester, Manchester, UK; ^7^Cambridge Stem Cell Institute, University of Cambridge, Cambridge, UK

##### **Correspondence:** Mary Goodwin - mag82@cam.ac.uk

*Fluids Barriers CNS* 2019, **16(2):** A16

**Introduction:** Cerebral Small Vessel Disease (SVD) causes 25% of stroke and 45% of dementia worldwide. Despite its prevalence, little is known about the mechanisms that cause or propagate the disease. However, neurovascular unit (NVU) dysfunction and perturbation of the matrisome are thought to play a key role in Blood–Brain Barrier (BBB) breakdown and SVD progression. Monogenic SVD caused by mutations in Collagen IV α1/α2 (COL4A1/2), a highly abundant matrisome protein, offer a tractable platform to study SVD pathophysiology in vitro.

**Methods:** Wild-type control (WT) and COL4A2G702D patient-derived human induced pluripotent stem cells (hiPSCs) were differentiated into brain microvascular endothelial cells (BMECs), astrocytes and mural cells, using previously published protocols. The cell types were characterised using qPCR, immunocytochemistry, flow cytometry and functional tests, including transendothelial electrical resistance (TEER), permeability to FITC-Dextran, LDL-uptake and tube formation. The three cell types were then combined into a Transwell-based co-culture model of the BBB.

**Results:** hiPSC-derived BMECs exhibit high TEER of 1500-2000 Ohm cm^2^, which is increased and maintained over two weeks in co-culture with astrocytes and in tri-culture with astrocytes and mural cells. WT and COL4A2G702D BMECs are impermeable to 10 kDa FITC-Dextran, uptake AF488-LDL and are capable of tube formation. Preliminary results from the COL4A2G702D SVD model suggest that COL4A2G702D BMECs exhibit increased expression of MMPs 2 and 9, relative to WT, as well as reduced Collagen IV deposition and lower tight junction protein expression.

**Conclusions:** The triple co-culture model offers a platform to assess the comparative functional capacity of the control and diseased cell types. Abnormal MMP expression and Collagen IV protein deposition are consistent with what is seen in patients and strengthens the argument that matrisome alterations could be important in COL4A1/2 SVD.

**Grant support:** This work was supported by the ACT-VAD (Stroke Association, British Heart Foundation, Alzheimer’s Society) and The Rosetrees Trust.

## A17 Glycine activation of NMDA Receptors on brain endothelial cells reduces barrier integrity mediated by increased intracellular calcium level

### Lisa Epping^1^, Manuela Cerina^1^, Lukas Gola^1^, Sjepana Kovac^1^, Juncal Fernandez-Orth^1^, Stefan Bittner^2^, Michael Platten^3,4^, Sven G. Meuth^1^

#### ^1^Department of Neurology with Institute for Translational Neurology, University Clinic Muenster, Muenster, Germany; ^2^Department of Neurology, University Medical Center Mainz, Mainz, Germany; ^3^DKTK CCU Neuroimmunology and Brain Tumor Immunology, German Cancer Research Center, Heidelberg, Germany; ^4^DKTK CCU Neuroimmunology and Brain Tumor Immunology, German Cancer Research Center, Heidelberg, Germany

##### **Correspondence:** Lisa Epping - lisa.epping@ukmuenster.de

*Fluids Barriers CNS* 2019, **16(2):** A17

In neurodegenerative disorders, concentrations of extracellular neurotransmitter in the central nervous system (CNS) are out of balance. Glutamatergic dysregulation and extracellular glycine accumulation cause excitotoxicity and subsequent neuronal death. This process is largely mediated by *N*-methyl-d-aspartate receptor (NMDAR) activation. NMDARs are ionotropic receptors permeable for positive ions. In the CNS, they are composed by two obligatory NR1 subunits containing glycine binding sites and two NR2 subunits that are activated by glutamate. NR3 subunits which are less abundant in the CNS are activated by glycine. While neuronal NMDARs are well studied, their functional role on endothelial cells (ECs) is still to be investigated. ECs of the Blood Brain Barrier (BBB) are exposed to the extracellular milieu of the CNS and therefore to high concentrations of neurotransmitters. In this study, we investigated the effect of NMDAR modulators on murine brain microvascular endothelial cells (MBMECs). Western blot experiments show expression of NR1, NR2a and NR3a subunits on MBMECs. Application of typical NR2 binding site activators (glutamate and quinolinic acid) did neither evoke any NMDAR mediated currents in electrophysiological experiments, nor affect Ca^2+^ levels. No changes in transendothelial electrical resistance (TEER) values of MBMECs were observed. However, glycine application to whole cell recorded MBMECs evoked a bigger NMDAR mediated current compared to control conditions and induced an increase in intracellular Ca^2+^ levels. Functionally, these changes were reflected in a reduced TEER and impaired MBMEC migration. Application of glycine but not glutamate binding site inhibitors rescued these effects. Taken together, these results suggest that glycine activated NMDARs composed of NR1 and NR3a subunits are expressed on MBMECs and that their activation triggers a loss in barrier function which is most likely mediated by intracellular Ca^2+^ changes.

**Grant Support:** The Groups of Sven G. Meuth and Stefan Bittner are funded by the “Transregional Collaborative Research Center (CRC) SFB TR-128 Initiating/effector versus regulatory mechanisms in Multiple Sclerosis - progress towards tackling the disease”; the Group of Sven G. Meuth and Michael Platten were funded by the DFG “FOR 2289: Kalzium-Homöostase bei Neuroinflammation und -degeneration: Neue Ansatzpunkte für die Therapie der multiplen Sklerose?”

## A18 T-cell migration across the BBB is regulated by endogenously produced AhR ligands under inflammatory conditions

### Lisa Epping^1^, Nadine Mykicki^2^, Jana Sonner^3^, Steffan Bittner^4^, Michael Platten^5,6^, Karin Loser^2^, Sven G. Meuth^1^

#### ^1^Department of Neurology with Institute for Translational Neurology, University Clinic Muenster, Muenster, Germany; ^2^Department of Dermatology, University of Münster, Münster, Germany; ^3^DKTK CCU Neuroimmunology and Brain Tumor Immunology, German Cancer Research Center, Heidelberg, Germany & Institute of Neuroimmunology and Multiple Sclerosis, University of Hamburg, Germany; ^4^Department of Neurology, University Medical Center Mainz, Mainz, Germany; ^5^DKTK CCU Neuroimmunology and Brain Tumor Immunology, German Cancer Research Center, Heidelberg, Germany; ^6^Department of Neurology, Medical Faculty Mannheim, Heidelberg University, Heidelberg, Germany

##### **Correspondence:** Lisa Epping - lisa.epping@ukmuenster.de

*Fluids Barriers CNS* 2019, **16(2):** A18

In the development of neuroinflammatory diseases, alterations of the blood brain barrier (BBB) represent key events. Factors that cause endothelial cells (ECs) of the BBB to develop an immune cell-permissive phenotype are poorly understood. It is known that EC-involving vascular dysfunction can be caused by kynurenine pathway (KP) metabolites. The enzyme indolamin-2,3-Dioxygenase (IDO-1) catalyzes the initial step of the KP in which tryptophan (TRP) is converted into various biologically active metabolites of which kynurneine and kynurenic acid are known to activate the aryl hydrocarbon receptor (AhR), a ligand binding transcription factor that mediates immune responses. In this study, we investigated the effect of IDO-1 induction and subsequent KP metabolite production on T-cell migration across murine brain microvascular endothelial cells (MBMECs) under inflammatory conditions. We could show that inflammation of MBMECs induced with IFNγ and TNFα results in IDO-1 expression and KP metabolite production which causes AhR activation and its translocation into the nucleus. Migration of T-cells across MBMEC monolayers from IDO-1−/− mice is reduced under inflammatory conditions in comparison to WT mice. The effect is mediated by a decrease in ICAM-1 expression. Interestingly, pharmacological inhibition of the AhR also results in a reduced T-cell migration across MBMEC monolayers from WT mice and it is accompanied by a decrease in ICAM-1 expression under inflammatory conditions. Additionally, these findings were confirmed in MBMECs obtained from AhR−/− mice, where a decreased T-cell migration under inflammation was also detected. Taken together, these results indicate a new endogenous mechanism in which IDO-1 activation through pro-inflammatory stimuli activates the AhR, which consequently mediates T-cell migration very likely being regulated by changes in ICAM-1 expression.

**Grant Support:** The Groups of Sven G. Meuth, Karin Loser and Stefan Bittner are funded by the “Transregional Collaborative Research Center (CRC) SFB TR-128 Initiating/effector versus regulatory mechanisms in Multiple Sclerosis - progress towards tackling the disease”; the Group of Sven G. Meuth and Michael Platten were funded by the DFG “FOR 2289: Kalzium-Homöostase bei Neuroinflammation und -degeneration: Neue Ansatzpunkte für die Therapie der multiplen Sklerose?”

## A19 Evaluation of Drug Disposition in Supratentorial Ependymoma

### Julia Benzel^1,2,3^, Max Sauter^5^, Norman Mack^1,2^, Abigail Davis^6^, Johanna Weiss^5^, Philipp Uhl^5^, Jürgen Burhenne^5^, Kendra K. Maass^1,2,4^, Jens-Martin Hübner^1,2,3^, Hendrik Witt^1,2,4^, Anang Shelat^7^, Amar Gajjar^8^, Aylin Camgoz^9^, Frank Buchholz^10,11,12,13,14^, Sina Oppermann^1,2^, Marcel Kool^1,2^, Daisuke Kawauchi^1,2^, Olaf Witt^1,2,4^, Walter Emil Haefeli^5^, Stefan M. Pfister^1,2,4^, Clinton Stewart^6^, Kristian W. Pajtler^1,2,4^

#### ^1^Hopp Children’s Cancer Center, NCT Heidelberg (KiTZ), Heidelberg, Germany; ^2^Division of Pediatric Neurooncology, German Cancer Consortium (DKTK), German Cancer Research Center (DKFZ), Heidelberg, Germany; ^3^Faculty of Biosciences, Heidelberg University, Heidelberg, Germany; ^4^Department of Pediatric Oncology, Hematology and Immunology, Heidelberg University Hospital, Heidelberg, Germany; ^5^Department of Clinical Pharmacology and Pharmacoepidemiology, Heidelberg University Hospital, Heidelberg University, Heidelberg, Germany; ^6^Department of Pharmaceutical Sciences, St. Jude Children’s Research Hospital, Memphis, Tennessee, USA; ^7^Department of Chemical Biology & Therapeutics, St. Jude Children’s Research Hospital, 262 Danny Thomas Place, Memphis, TN 38105, USA; ^8^Department of Oncology, St. Jude Children’s Research Hospital, Memphis, TN, 38105, USA; ^9^Medical Faculty and University Hospital Carl Gustav Carus, UCC Section Medical Systems Biology, TU Dresden, 01307 Dresden, Germany; National Center for Tumor Diseases (NCT), University Hospital Carl Gustav Carus, TU Dresden, 01307 Dresden, Germany; ^10^Medical Faculty and University Hospital Carl Gustav Carus, UCC Section Medical Systems Biology, TU Dresden, 01307 Dresden, Germany; ^11^German Cancer Research Center (DKFZ), Heidelberg; ^12^German Research Consortium (DKTK), Partner Site Dresden, 01307 Dresden, Germany; 13National Center for Tumor Diseases (NCT), University Hospital Carl Gustav Carus, TU Dresden, 01307 Dresden, Germany; ^14^Max Planck Institute of Molecular Cell Biology and Genetics, 01307 Dresden, Germany

##### **Correspondence:** Julia Benzel - j.benzel@kitz-heidelberg.de

*Fluids Barriers CNS* 2019, **16(2):** A19

The majority of supratentorial (ST) ependymomas (EPN) in children is driven by distinct gene fusions between the partners C11orf95 and RELA. Tumors belonging to the resulting molecular group ST-EPN-RELA are characterized by activation of NF-κB signaling and deregulation of the p53 pathway. In contrast to surgery and radiotherapy, chemotherapeutic concepts have so far failed in the clinical management of these tumors. Using RNAi and drug screening methods, we identified p53 reactivation and inhibition of nuclear protein shuttling as promising therapeutic approaches. While low dose dactinomycin can successfully reestablish p53 function in ST-EPN-RELA cells, penetration of the drug through the blood–brain barrier (BBB) is poor. We are currently exploring alternative strategies such as combination with efflux pump inhibitors or liposomal packaging to overcome these constraints. In addition, idasanutlin, a brain penetrant MDM2 inhibitor, and selinexor, a covalent, slowly reversible, and selective inhibitor of XPO1 being the sole nuclear exporter of p53, have entered preclinical in vivo studies in ST-EPN-RELA models. Inhibition of XPO1 also enhances nuclear localization of IκBα, which is expected to result in downregulation of NF-κB transcriptional activity in ST-EPN-RELA cells. In order to identify optimal dosing strategies and assess effects of combinatorial treatment approaches on BBB penetration, we here apply cerebral microdialysis combined with ultraperformance liquid chromatography coupled to tandem mass spectrometry (UPLC-MS/MS) analyses, which allows exact, continuous, and time-dependent drug quantification in tumors or healthy tissue in freely-moving experimental animals.

## A20 Whether the choroid plexuses from the brain ventricles differ in the genes expression?

### Aleksandra Szczepkowska, Sylwia Buczkowska, Anna Sawinska, Janina Skipor

#### Institute of Animal Reproduction and Food Research, Polish Academy of Sciences, Olsztyn, Poland

##### **Correspondence:** Aleksandra Szczepkowska - a.szczepkowska@pan.olsztyn.pl

*Fluids Barriers CNS* 2019, **16(2):** A20

**Introduction:** Choroid plexuses (CPs) are localized in the brain ventricles (the third, two lateral and the fourth) and form interface between the blood and the cerebrospinal fluid (CSF). They are composed of tightly connected epithelial cells, mainly responsible for active CSF production, surrounding a core in which blood vessels are fenestrated. Proper CSF production is dependent on: (1) maintaining of fenestration of CP capillaries (vascular endothelial growth factor/receptors—VEGF/VEGFR) and water transport through CP epithelium (aquaporin 1—AQP1, transcellular and claudin 2—CLDN2, paracellular passage). This study evaluates mRNA expression of mentioned above factors in the ovine CPs collected from different ventricles.

**Methods:** Studies were performed on adult ewes (4–5 years old, n = 12) during the period of increasing days (April). CPs were collected separately from the fourth ventricle and from the third ventricle together with the lateral ventricles immediately after sacrifice at 3 different time points: 11:00 (n = 4), 16:00 (n = 4), and 22:00 (n = 4). The real-time PCR analysis of mRNA expression of VEGF isoforms (VEGF164 and VEGF120) and its receptors (VEGFR-1 and VEGFR-2), AQP1 and CLDN-2 were performed.

**Results:** The significantly (p < 0.05) higher expression of VEGF164 in all time points, and VEGF120 at 16:00, was observed for CPs from fourth compared to lateral ventricles. The expression of VEGFRs did not differ between CP types. The expression of AQP1 was significantly (p < 0.05) lower in CPs from fourth compare to lateral ventricles at 2 time points: 11:00 and 22:00, and showed common tendency (p = 0,.54) at 16:00. The expression of CLDN2 was slightly lower (p = 0.067) in CPs from fourth compare to lateral ventricles, but only at 16:00.

**Conclusion:** It seems to be important to consider CPs from the fourth and lateral ventricles, separately when studying VEGF, AQP1 and CLDN2.

**Grant support:** This work was supported by KNOW (Leading National Research Centre) Scientific Consortium “Healthy Animal - Safe Food”, decision of Ministry of Science and Higher Education No. 05-1/KNOW2/2015.

## A21 Investigating the Integrity of the Blood–Brain Barrier in Neuropsychiatric Disorders

### Nicole Hanley, Chris Greene, Matthew Campbell

#### Smurfit Institute of Genetics, Trinity College Dublin, Dublin, Ireland

##### **Correspondence:** Nicole Hanley - nihanley@tcd.i.e

*Fluids Barriers CNS* 2019, **16(2):** A21

**Introduction:** The blood–brain barrier (BBB) is a physical barrier formed by endothelial cells of the central nervous system and cells of the neurovascular unit. Transport across the barrier is tightly regulated by the presence of tight junctions (TJs) composed of a number of transmembrane proteins including claudins, occludin and junctional adhesion molecules. Claudin-5 is of particular importance in maintaining integrity of the BBB and changes in its expression has been implicated in a number of disorders including stroke, traumatic brain injury and psychiatric disorders such as schizophrenia. The aim of this study was to assess the integrity of the blood–brain barrier in a cohort of 60 postmortem brain samples from the Stanley Medical Research Institute, including 15 bipolar disorder, 15 major depression, 15 schizophrenia and 15 unaffected controls.

**Methods:** The integrity of the blood–brain barrier was analysed by immunohistochemical (IHC) analysis of fresh frozen brain sections from the hippocampus and orbital frontal cortex. Changes in transcript levels of TJ proteins were assessed by qPCR of RNA isolated from the caudal cingulate, cerebellum, parietal, occipital and premotor cortex.

**Results:** IHC analysis of claudin-5 protein levels revealed decreased claudin-5 expression in the hippocampus of major depression and schizophrenia. RNA transcript levels of claudin-5 were upregulated in the cerebellum and occipital cortex of bipolar disorder. Correlation analysis looking at age of onset or duration of each disease versus TJ expression were performed and showed negative correlations between levels of TJ proteins and duration of bipolar disorder across multiple brain regions.

**Conclusion:** Here we showed that the integrity of the BBB has been compromised in a cohort of patients with neuropsychiatric disorders. This suggests an important role of BBB disruption in the development and prognosis of each disorder that could be targeted therapeutically in the future.

**Grant support:** This work was supported by the Science Foundation Ireland (SFI), Health Research Board (HRB), Irish Research Council (IRC).

## A22 Characterization of AAV to restore Claudin-5 expression at the Blood Brain Barrier and its therapeutic potential in schizophrenia

### Claire O’Connor, Chris Greene, Matthew Campbell

#### Smurfit Institute of Genetics, Trinity College Dublin, Dublin, Ireland

##### **Correspondence:** Claire O’Connor - oconnc43@tcd.i.e

*Fluids Barriers CNS* 2019, **16(2):** A22

The blood brain barrier (BBB) positioned along blood vessels of the central nervous system (CNS) is a highly selective and tightly regulated barrier. The principal components of the BBB are endothelial cells (EC), which have developed numerous specialisations which limit movement of material from blood to brain and vice versa. Paracellular transport is restricted due to the presence of tight junctions (TJ), dynamic structures linking adjacent ECs. Claudins, a family of transmembrane proteins, act as the main determinants of TJ properties, with claudin-5 (CLDN5) being the most enriched. Previous work in the lab has identified a link between CLDN5 suppression and psychosis through an inducible knockdown mouse model.

Schizophrenia is a neurodevelopmental disorder that affects almost 1% of the population. However, in patients with 22q11 deletion syndrome (22q11DS), this prevalence rises to 30%. Due to a microdeletion within the 22q11.21 chromosomal region, this population is haploinsufficent for the CLDN5 gene. Importantly, CLDN5 is a dosage sensitive gene that also has polymorphisms which result in reduced gene expression. The lab has previously demonstrated that the reduced level of CLDN5 expression that results from carrying both a low expression allele and being haploinsufficent, may predispose individuals to schizophrenia.

Therefore, our main objective is to determine if restoring CLDN5 expression at the BBB prevents a schizophrenia like phenotype. To do this, we will use an adeno-associated viral (AAV) vector to increase CLDN5 expression. As the overall aim is to translate this molecular modulation strategy into a clinical setting, the efficacy of the AAV must be optimised. This has been done by screening various plasmids in vitro, examining changes in gene expression and its temporal nature, the effect of promoter specificity and the use of inducible expression systems. Their effects on the expression of CLDN5 in vitro was investigated by IHC, qPCR and WB analysis.

**Grant support:** This work was supported by the Irish Research Council Laureate Awards Programme 2017/2018.

## A23 Imaging the central nervous system immune privilege: The role of the brain barriers in regulating trafficking of immune cells and immune mediators in the central nervous system

### Josephine Angelo Mapunda, Mykhailo Vladymyrov, Britta Engelhardt

#### University of Bern, Theodor Kocher Institute, Bern, Switzerland

##### **Correspondence:** Josephine Angelo Mapunda - josephine.mapunda@tki.unibe.ch

*Fluids Barriers CNS* 2019, **16(2):** A23

**Introduction:** The central nervous system (CNS) is an immune privileged site. The brain barriers separate the CNS into compartments that significantly vary with respect to their accessibility to immune cell subsets and their drainage of antigens from the CNS to the peripheral immune system. It has been shown that the blood–brain barrier (BBB) and blood cerebrospinal fluid barrier (BCSFB) can be breached by activated immune cells to reach the cerebrospinal fluid (CSF) filled ventricular and subarachnoid spaces, allowing for CNS immune surveillance without the risk of disturbing homeostasis of the CNS parenchyma, which is lined by the glia limitans. Recent studies have proposed the existence of a glymphatic system in the CNS where CSF mixes with the interstitial fluid (ISF) of the CNS parenchyma and is drained via dural lymphatic vessels into peripheral lymph nodes. These concepts ignore the existence of the brain barriers that may prohibit such a fluid exchange.

**Methods:** We here propose that disturbance of the brain barriers may cause CNS pathology and thus disruption of the CNS immune privilege. To investigate this hypothesis, we are using and establishing novel reporter mice allowing for visualization of the brain barriers by two photon intravital microscopy in the brain and spinal cord of mice during health and in animal models of multiple sclerosis and Alzheimer’s Disease. This approach will allow us to visualize the distribution of cellular and humoral factors of the immune system in the different CNS compartments bordered by the brain barriers.

**Grant support:** This work was supported by the Swiss Government Excellence Scholarship; FBRI.

## A24 In vitro identification of cell-surface proteins internalized into human brain microvascular endothelial cells using SWATH-based quantitative proteomics

### Shingo Ito^1^, Mariko Oishi^1^, Tatsuki Uemura^1^, Takeshi Masuda^1^, Tomomi Furihata^2^, Pierre-Olivier Couraud^3^, Sumio Ohtsuki^1^

#### ^1^Kumamoto University, Kumamoto, Japan; ^2^Tokyo University of Pharmacy and Life Sciences, Tokyo, Japan; ^3^Institute Cochin, Paris, France

##### **Correspondence:** Shingo Ito - ishingo@kumamoto-u.ac.jp

*Fluids Barriers CNS* 2019, **16(2):** A24

**Introduction:** Receptor-mediated transcytosis (RMT) is the potential method for transferring biopharmaceuticals across the blood–brain barrier (BBB), and the cell-surface proteins, internalized into brain capillary endothelial cells, serve as promising candidates for this process. The present study aimed to identify cell-surface proteins internalized into human brain microvascular endothelial cells using surface biotinylation method and SWATH-based quantitative proteomics.

**Methods:** After biotinylation of cell-surface proteins, an internalization study was performed with 20% human serum. The biotinylated proteins internalized into the cells were recovered by streptavidin beads, followed by identification and quantification by SWATH-based quantitative proteomics.

**Results:** Using SWATH-MS-based quantitative proteomics, 378 cell-surface biotinylated proteins were identified in hCMEC/D3 cells, a model of human brain microvascular endothelial cell, and 125 proteins were identified to be internalized into hCMEC/D3. Compared to identification and expression levels of proteins in HUVEC, a model of peripheral microvascular endothelial cell, 35 selectively internalized proteins were identified in hCMEC/D3 cells. RNAseq database analysis showed 10 proteins with high mRNA expression in human brain endothelial cells, and 8 of these 10 proteins showed high levels of internalization into hCEMC/D3 cells. These 8 proteins were expressed in the plasma membrane fraction of HBMEC/Cibeta another model of human brain microvascular endothelial cell, as well as in hCMEC/D3. Based on immunohistochemistry-based atlas of protein expression profiles in normal tissues, 6 of the 8 proteins were at least localized in human brain microvessels.

**Conclusion:** We identified 6 cell-surface proteins selectively internalized into human brain microvascular endothelial cells, and our findings provide novel strategies for the development of a BBB-permeable drug delivery system.

**Grant support:** This work was supported by the AMED, JST-CREST, Grant-in-Aid for Scientific Research (B).

## A25 Protection against the damaging effects of kainate: a study on a culture model of the blood–brain barrier

### Lilla Barna^1^, Fruzsina Walter^1^, András Harazin^1^, Alexandra Bocsik^1^, Vilmos Tubak^2^, Katalin Jósvay^3^, Patricia Campos-Bedolla^4^, Mária A Deli^1^

#### ^1^Institute of Biophysics, Biological Research Centre of the Hungarian Academy of Sciences, Szeged, Hungary; ^2^Creative Laboratory Ltd., Szeged, Hungary; ^3^Institute of Biochemistry, BRC HAS, Szeged, Hungary; ^4^Unidad de Investigacion Medica en Enfermedades Neurologicas, Hospital de Especialidades, Centro Medico Nacional Siglo XXI, Instituto Mexicano del Seguro Social, Mexico, D.F., Mexico

##### **Correspondence:** Lilla Barna - barna.lilla@brc.mta.hu

*Fluids Barriers CNS* 2019, **16(2):** A25

In animal models of epilepsy induced by kainic acid, the primary ligand of ionotropic kainate receptors, BBB damage occurs, but the effect of kainate on brain endothelial cells has not been studied. Our aim was to examine the presence of kainate receptors and the effects of kainate on cultured cells of the BBB and to test anti-inflammatory and antioxidant drugs to protect against kainate-induced changes. Rat primary brain endothelial, pericyte and astroglial cell cultures were used to study cell viability by impedance measurement and cell morphology. BBB permeability was measured on a model made from the three cell types. The production of nitrogen monoxide and reactive oxygen species was followed by fluorescent probes. Four drugs were tested to protect against kainate-induced changes. The mRNA expression of kainate receptors and nitric oxide synthases (NOS) were studied by RT-PCR. The presence of kainate receptors was demonstrated on brain endothelial cells by PCR. Kainate damaged brain endothelial cells in a concentration- and time-dependent way and made the immunostaining of claudin-5 discontinuous at the cell border indicating the opening of the barrier. The viability or the morphology of astroglial cells were unchanged. Kainic acid increased the permeability of the BBB model for marker molecules and the production of nitric oxide (NO) in brain endothelial cells. Dexamethasone, simvastatin and edaravone protected against the reduced cell viability and increased permeability caused by kainate. Dexamethasone attenuated the elevated NO production and iNOS/NOS2 mRNS expression caused by kainate treatment. In conclusion, we demonstrated the presence of kainate receptors on brain endothelial cells and that kainate directly damages brain endothelial cells. Our results suggest that kainate receptors of brain endothelial cells could be therapeutic targets in epilepsy to attenuate BBB damage.

**Grant support:** This work was supported by the bilateral grant from the Hungarian Academy of Sciences.

## A26 In vitro involvement of creatine transporter in the influx of cyclocreatine into HEK293 and hCMEC/D3 cells

### Tatsuki Uemura^1^, Shingo Ito^1^, Takeshi Masuda^1^, Pierre-Olivier Couraud^2^, Sumio Ohtsuki^1^

#### ^1^Kumamoto University, Kumamoto, Japan; ^2^Institute Cochin, Paris, France

##### **Correspondence:** Tatsuki Uemura - 176y2005@st.kumamoto-u.ac.jp

*Fluids Barriers CNS* 2019, **16(2):** A26

**Introduction:** Creatine transporter (CRT) is involved in supplying creatine to neurons across the blood–brain barrier (BBB). Deletion of CRT causes cerebral creatine deficiency syndromes (CCDSs). Cyclocreatine, a creatine analog, may be a promising drug for treating CCDS patients. However, whether cyclocreatine is a substrate for CRT or not, and the transporters involved in its transport across the BBB remain unclear. This study aimed to elucidate the involvement of CRT in cyclocreatine transport using 2 cell lines. One line is HEK293 cells, which possess endogenous CRT-mediated creatine uptake, and the other is hCMEC/D3 cells, which are immortalized human brain microvascular endothelial cells.

**Methods:** Cells were incubated with [14C]cyclocreatine (9 µM) and [14C]creatine (9 µM) for the indicated times at 37 °C in the presence or absence of several compounds. siRNAs were transfected by lipofection. Protein and mRNA expressions were quantified by LC–MS/MS and qPCR, respectively.

**Results:** [14C]cyclocreatine was taken up in a time-dependent manner by HEK293 cells, and exhibited saturable kinetics, with a Km of 188 µM and Vmax of 273 pmol/mg protein/min. The uptake of [14C]cyclocreatine by HEK293 cells was inhibited 97% by 10 mM unlabeled creatine, and 96% by 10 mM guanidinopropionic acid (GPA), a specific inhibitor of CRT. Treatment of HEK293 cells with CRT-targeting siRNA reduced CRT protein expression by 42%, and inhibited uptake of [14C]cyclocreatine by 33% and [14C]creatine by 37%. In hCMEC/D3 cells, uptake of [14C]cyclocreatine was suppressed by 91%, both by unlabeled creatine and GPA. Treatment of hCMEC/D3 cells with CRT-targeting siRNA reduced CRT mRNA expression by 97%, and inhibited uptake of [14C]cyclocreatine by 70% and [14C]creatine by 63%.

**Conclusion:** The present results suggest that cyclocreatine is a CRT substrate; CRT at the BBB is the predominant contributor to influx transport of cyclocreatine into the brain. Our findings provide important insights for treating CCDS patients with cyclocreatine.

**Grant support:** This work was supported by AMED, Grant-in-Aid for Scientific Research (B).

## A27 Generation of disease specific induced pluripotent stem cells into a blood–brain barrier system for the analysis of APOE4 and its role in Alzheimer’s disease

### Carla Hartmann^1^, Jenny Pfeifer^1^, Toni Ehrhardt^1^, Annette Hartmann^1^, Ina Giegling^1^, Undine Haferkamp^2^, Ole Pless^2^, Winfried Neuhaus^3^, Dan Rujescu^1^ and Matthias Jung^1^

#### ^1^University and Outpatient Clinic for Psychiatry, Psychotherapy, and Psychosomatic Medicine, Martin Luther University Halle-Wittenberg, Halle/Saale, Germany; ^2^Biomarker and Translational Drug Discovery Fraunhofer IME ScreeningPort, Hamburg, Germany; ^3^AIT Austrian Institute of Technology, Department Health and Environment, Molecular Diagnostics, Vienna, Austria

##### **Correspondence:** Carla Hartmann - carla.hartmann@uk-halle.de

*Fluids Barriers CNS* 2019, **16(2):** A27

**Introduction:** One pathological characteristic of late-onset Alzheimer’s disease (AD) is the accumulation of amyloid-β (Aβ) peptides. Dysregulation of the blood–brain barrier (BBB) confers the risk for suffering from AD and contributes to disease progression. The most associated genetic risk factor is the E4 allele of apolipoprotein E (APOE). APOE is involved in a number of metabolic pathways including lipid transport, Aβ aggregation, and Aβ clearance. However, the molecular and cellular signaling pathways regulated by APOE4 are currently poorly understood. The differentiation of BBB cells using patient-derived induced pluripotent stem cells (iPSCs) is a promising approach to analyze AD disease mechanisms in the context of APOE4 and its role in BBB breakdown.

**Methods:** First, we identified the APOE status of our AD patients and healthy matched controls. Then, episomal vectors were used for the generation of iPSCs from B-lymphoblastoid cell lines of patients carrying the APOE4 allele and healthy donors with homozygous APOE3 alleles. Following the verification of pluripotency the established iPSCs were used for differentiation into cells of the BBB system. Expression levels of BBB specific markers and barrier functionality were analyzed.

**Results:** After successful generation of AD-specific iPSCs and proven pluripotency, we differentiated them into cells of the BBB system, in particular endothelial cells and astrocytes. Efficient differentiation was shown by the expression of cell- and BBB-specific markers including OCLN and TJP1. Barrier functionality was demonstrated by hindered paracellular transport of sodium fluorescein and transendothelial electrical resistance values > 1000 Ohm*cm2 for both AD patients and matching controls.

**Conclusion:** Overall, we established a patient- and disease-specific BBB model suitable to investigate AD-associated genetic risk variants, resulting pathogenic phenotypes, and underlying disease mechanisms providing potential targets for AD treatment.

**Grant support:** This work was supported by the HiPSTAR, BMBF (01EK1608C).

## A28 Blood–brain barrier changes in acute pancreatitis: a study using cell culture models

### Fruzsina Walter^1^, András Harazin^1^, Szilvia Veszelka^1^, Andrea E Toth^1^, Nora Horanyi^1^, Ana R. Santa-Maria^1^, Balazs Kui^2^, Zsolt Balla^2^, József Maléth^2^, Vilmos Tubak^3^, Agnes Kittel^4^, Péter Hegyi^5,6^, Zoltan Rakonczay^2^, Maria A Deli^1^

#### ^1^Biological Research Centre of the Hungarian Academy of Sciences, Szeged, Hungary; ^2^Institute of Pathophysiology, University of Szeged, Szeged, Hungary; ^3^Creative Laboratory Ltd., Szeged, Hungary; ^4^Institute of Experimental Medicine, Hungarian Academy of Sciences, Budapest, Hungary; ^5^First Department of Medicine, University of Szeged, Szeged, Hungary; ^6^Institute of Translational Medicine, University of Pécs, Pécs, Hungary

##### **Correspondence:** Fruzsina Walter **-** walter.fruzsina@brc.mta.hu

*Fluids Barriers CNS* 2019, **16(2):** A28

Acute pancreatitis (AP) is a serious inflammatory disease with excessive production and failed secretion of enzymes in the pancreas accompanied by edema, local cell death which may progress to systemic inflammation and organ failure. The central nervous system is affected in 10% of all severe AP cases manifesting in pancreatic encephalopathy, with an even higher mortality. The development of pancreatic encephalopathy is not well understood. Earlier research from our laboratory described for the first time that during the course of taurocholate induced pancreatitis blood–brain barrier (BBB) permeability is elevated in rats. These results were corroborated by using a rat non-invasive acute pancreatitis model induced by the administration of l-ornithine. This treatment induced elevated vessel permeability in the pancreas and the brain, along with the damage of glycocalyx in the vascular endothelium. Our data indicate that BBB morphology and barrier functions are damaged in this rat model of AP. Therefore, we aimed to explore the mechanisms, factors and cellular interactions involved in BBB dysfunction in AP by using a rat primary brain endothelial co-culture model in static conditions and under dynamic settings by using a cutting-edge biochip model of the BBB (Walter et al., 2016, 10.1016/j.snb.2015.07.110). We found, that l-ornithine treatment decreases cell impedance and elevates permeability after 24 h treatment. We have shown morphological and expression changes for several key interendothelial junctional and adhesion molecules after l-ornithine treatment. Along with these observations reactive oxygen species production was increased and mitochondrial network was damaged. In conclusion, in this model of AP changes in BBB functions could be observed. Our results may help to better understand the background of BBB alterations during AP and could lead to the development of future treatment strategies.

**Grant support:** F.R.W. is supported by the János Bolyai Research Fellowship of the Hungarian Academy of Sciences, by the National Research, Development and Innovation Office, Hungary [grant number OTKA PD-128480] and by the Richter Gedeon Pharmaceutical Company, Budapest.

## A29 Validation of a novel cell model (iP-gp) expressing human P-glycoprotein; Comparisons of in vitro efflux ratios obtained in the iP-gp cell model with corresponding efflux rations ratios from MDCK-MDR1 cells and Kp,uu values from mice

### Burak Ozgur^1^, Lasse Saaby^2^, Kristine Langthaler^3^, Elin Eneberg^3^, Christian Janfelt^1^, Lassina Badolo^4^, Dino Montanari^3^ and Birger Brodin^1^

#### ^1^Department of Pharmacy, University of Copenhagen, Copenhagen, Denmark; ^2^Bioneer:FARMA, Bioneer A/S, Copenhagen, Denmark; ^3^H. Lundbeck A/S, Copenhagen, Denmark; ^4^Merck KGaA, Darmstadt, Germany

##### **Correspondence:** Burak Ozgur - burak.ozgur@sund.ku.dk

*Fluids Barriers CNS* 2019, **16(2):** A29

P-glycoprotein (P-gp, ABCB1) is considered as one of the major obstacles to drug delivery into the brain parenchyma. Cell models expressing human P-gp are often used for screening of new drug candidates during early states of drug development. It is however uncertain how well the in vitro studies can predict the in vivo relevance of P-gp mediated efflux at the BBB. The present study aimed at evaluating the applicability of the human P-gp expressing iP-gp cell line for screening new drug candidates for their susceptibility to P-gp interactions in bidirectional transport experiments, and how well the cell line could predict the in vivo relevance of P-gp mediated efflux at the BBB in mice. For this purpose, the bidirectional fluxes of 14 drug candidates were measured in iP-gp cells and in MDCK II MDR1 cells, and compared with pharmacokinetic data obtained in male C57BL/6 mice. The iP-gp cells formed extremely tight monolayers (> 15 000 Ohm cm^2^) as compared to the MDCK II MDR1 cells (> 250 Ohm cm^2^), resulting in generally lower Papp in the apical to basolateral (A-B) direction. Both cell models were capable of discriminating between compounds with low and high permeation. The efflux ratios obtained with iP-gp and MDCK-MDR1 monolayers correlated inversely with Kp,uu values from the in vivo studies, where compounds with the lowest Kp,uu generally displayed the highest efflux ratios. 12 of the tested compounds displayed a poor BBB penetration in mice as judged by Kp,uu less than 1, indicating a possible involvement of an efflux transporter. Of these compounds, seven compounds were identified as P-gp substrates in the iP-gp screen, whereas analysis of data from the MDCK II MDR1 cell indicated four compounds were substrates. This suggests that the iP-gp cell model may be a sensitive and useful screening tool to identify possible substrates of human P-glycoprotein.

**Grant support:** This work was supported by the Lundbeck Foundation Research Network on Brain Barriers and Drug Delivery (RIBBDD).

## A30 Dynamic tight junction remodelling in epilepsy; the role of claudin-5

### Chris Greene^1^, Nicole Hanley^1^, Cristina Reschke^2^, Eoin O’Keeffe^1^, Natalie Hudson^1^, Carme Vila Sala^3^, Eoin Kelly^4^, Colin Doherty^5^, Matthew Campbell^1^

#### ^1^Trinity College Dublin, Dublin, Ireland; ^2^Royal College of Surgeons Ireland, Dublin, Ireland; ^3^Beaumont Hospital Dublin, Dublin, Ireland; ^4^St James’s Hospital Dublin, Dublin, Ireland; ^5^Beaumont Hospital Dublin, Dublin, Ireland

##### **Correspondence:** Chris Greene - greenech@tcd.i.e

*Fluids Barriers CNS* 2019, **16(2):** A30

**Introduction:** Epilepsy is characterised by recurrent seizure events mediated by abnormal neural activity in the central nervous system. At the blood–brain barrier (BBB), it is known that leakage of serum components into the brain is associated with the severity of epilepsy and is an early event in the pathogenesis of the disease. We and others have previously shown that knockdown of the tight junction protein claudin-5 in the adult rodent brain is associated with psychosis, depression and inflammation due to increased BBB permeability.

**Results:** Here, we show that loss of claudin-5 occurs early in the kainic acid model of temporal lobe epilepsy. Vascular leakage of Sulfo-NHS-biotin and FITC-dextran 70 kDa in the mouse brain occurs in hippocampal blood vessels characterised by weak and discontinuous tight junction staining for claudin-5 and zo-1. Acutely seizing and chronic spontaneously seizing mice have significantly elevated levels of serum S100B and VEGF. Electroencephalographic (EEG) analysis revealed convulsive seizures in mice following persistent suppression of claudin-5 in a doxycycline-inducible knockdown model. These events were concomitant with neurovascular unit remodelling including vascularisation and activation of astrocytes and microglia in hippocampal regions. In humans with drug resistant epilepsy, BBB disruption was evident following dynamic contrast enhanced MRI (DCE-MRI). Additionally, sections of resected temporal lobe from these patients showed widespread disruption of claudin-5 and zo-1, leakage of immunoglobulin, fibrinogen and albumin and, interestingly, widespread deposition of perivascular hyperphosphorylated tau in regions devoid of claudin-5 immunoreactivity.

**Conclusions:** Overall, our data indicate that loss of claudin-5 is an important event during the pathogenesis of seizure activity in vivo. Therapeutically targeting the brain vasculature to restore BBB integrity may be promising approach to alleviate the burden of epilepsy.

**Grant support:** This work was supported by the Health Research Board, Irish Research Council, FutureNeuro.

## A31 SensorTransBBB - Establishment of a Microfluidic Chip Model of the Blood–Brain-Barrier

### Sebastian Trennheuser^1^, Felix Schmitt-Hoffner^1^, Julia Botta^1^, Martin Stelzle^2^, Gert Fricker^1^

#### ^1^Department of Pharmaceutical Technology, Institute of Pharmacy and Molecular Biotechnology, Heidelberg University, Im Neuenheimer Feld 329, 69120 Heidelberg, Germany; ^2^NMI Natural and Medical Sciences Institute at the University of Tübingen, Markwiesenstrasse 55, 72770 Reutlingen, Germany

##### **Correspondence:** Sebastian Trennheuser - s.trennheuser@uni-heidelberg.de

*Fluids Barriers CNS* 2019, **16(2):** A31

**Introduction:** The human blood–brain-barrier (BBB) is a highly selective cellular structure which separates the central nervous system (CNS) from the circulating blood stream. It plays a very important role for the protection of the CNS and therefore prevents a lot of pharmaceutical drugs from reaching the brain. Furthermore, a variety of diseases of the CNS are associated with dysfunctions of the BBB. In order to investigate the barrier functions of the BBB and drug transport under physiological and pathophysiological conditions, the SensorTransBBB system was developed. The microphysiological chip in microwell format, produced by micro-injection molding, consists of ten parallel culture channels which can be perfused with cell culture medium.

**Methods:** Porcine brain capillary endothelial cells (PBCECs) were cultivated on the surface of a synthetic hydrogel matrix. Important cell culture conditions like seeding density, attachment time, medium volume etc. were determined in order to obtain a dense cell layer under perfusion. The viability of PBCECs was assessed by calcein stainings and cell layer integrity was determined by a permeability assay using FITC dextran (20 kDa). Furthermore, the tight junction protein ZO 1 was demonstrated by immunostainings.

**Results:** PBCECs formed a tight cell layer on the hydrogel surface, preventing FITC dextran from permeating the cellular barrier. The presence of ZO-1 at the cell borders was shown, indicating a functional BBB. Furthermore, the perfusion experiments suggested the critical role of medium volume for cell layer integrity under perfusion.

**Conclusion:** The SensorTransBBB Chip provides a promising, scalable microfluidic platform for the investigation of cellular barriers, which can be integrated seamlessly into existing cell culture and drug testing workflows.

**Grant support:** This work was supported by the Baden-Württemberg Stiftung, grant no. MIVT_002; BMBF, grant no. FKZ16SV5952.

## A32 Adaptation of the human in vitro blood–brain barrier model for the analysis of serum-derived factors in breast cancer patients with brain metastases

### Carolin J. Curtaz^1^, Constanze Schmitt^2^, Achim Woeckel^1^, Norbert Roewer^2^, Fabien Gosselet^3^, Malgorzata Burek^2^

#### ^1^University of Würzburg, Department of Gynecology and Obstetrics, Würzburg, Germany; ^2^University of Würzburg, Department of Anaesthesia and Critical Care, Würzburg, Germany; ^3^Blood-Brain Barrier Laboratory, University d’Artois, Lens, France

##### **Correspondence:** Carolin Julia Curtaz - curtaz_c@ukw.de

*Fluids Barriers CNS* 2019, **16(2):** A32

**Introduction:** In vitro blood–brain barrier (BBB) models serve as a useful tool to study the molecular mechanisms behind brain pathologies. Circulating tumor cells must pass through the BBB before forming cerebral metastases. The underlying mechanism of this process is largely unknown. In this study, we adapted an in vitro human BBB model to investigate the effects of serum-derived factors in breast cancer patients with brain metastases on BBB integrity in vitro.

**Methods:** The in vitro human BBB model is based on endothelial cells differentiated from the CD34+ hematopoietic stem cells of umbilical cord blood. Human brain-like endothelial cells (BLECs) were cultured with bovine pericytes for 6 days to induce BBB-like properties. Expression of marker proteins was analyzed by Western blot and immunostaining. The study protocol for collecting patient samples was approved by the local ethics committee and written consent was obtained from the patients. Serum samples were taken from breast cancer patients with brain metastases and a control group. Extracellular vesicles (EVs) were isolated from patient serum. The EVs serum concentration was measured by CD63-ELISA. The permeability of fluorescein-Na was used to estimate barrier integrity.

**Results:** The staining of BLECs cultured with pericytes showed high claudin-5 and zonula occludens-1 expression at the tight junctions. In addition, co-culture with pericytes induced the expression of junctional proteins, transporters and cellular receptors in BLECs. This correlated with the low permeability for the paracellular marker fluorescein (400 Da). The levels of CD63-positive EVs in serum were similar in both study groups of patients.

**Conclusion:** CD34+ cell-derived human in vitro BBB model exhibits high barrier properties, accompanied by the expression of BBB and endothelial cell markers. It is being used in ongoing work to study the mechanisms underlying the formation of brain metastasis in breast cancer.

## A33 Loud music/sound opens the blood–brain barrier suggesting a readily available approach to brain drug delivery and therapy in brain diseases

### Oxana Semyachkina-Glushkovskaya^1^, Vladimir Chekhonin^2^, Denis Bragin^1,3^, Olga Bragina^1,3^, Elena Vodovozova^4^, Anna Alekseeva^4^, Vladimir Salmin^5^, Andrey Morgun^5^, Natalia Malinovskaya^5^, Elena Osipova^5^, Elizaveta Boytsova^5^, Abolghasem Tohidpour^5^, Alexander Shirokov^6^, Nikita Navolokin^7^, Yirong Yang^8^, Chao Zhang^9,10^, Wei Feng^9,10^, Arkady Abdurashitov^1^, Alexander Khorovodov^1^, Andrey Terskov^1^, Ali Esmat Shareef^1^, Maria Klimova^1^, Ilana Agranovich^1^, Aysel Mamedova^1^, Alexy Pavlov^1^, Qingming Luo^9,10^, Dan Zhu^9,10^, Ivan Fedosov^1^, Anton Namikin^1^, Valery Tuchin^1,11,12^, Jürgen Kurths^1,13,14^

#### ^1^Saratov State University, Saratov, Russian Federation; ^2^Russian National Research Medical University, Moscow, Russian Federation; ^3^University of New Mexico School of Medicine, Albuquerque, USA; ^4^Shemyakin-Ovchinnikov Institute of Bioorganic Chemistry, Russian Academy of Sciences, Moscow, Russian Federation; ^5^Krasnoyarsk State Medical University, Krasnoyarsk, Russian Federation; ^6^Institute of Biochemistry and Physiology of Plants and Microorganisms, Russian Academy of Sciences, Saratov, Russian Federation; ^7^Saratov State Medical University, Saratov, Russian Federation; ^8^University of New Mexico, College of Pharmacy, Albuquerque, USA; ^9^Wuhan National Laboratory for Optoelectronics-Huazhong University of Science and Technology, Wuhan, China; ^10^MoE Key Laboratory for Biomedical Photonics, Collaborative Innovation Center for Biomedical Engineering, School of Engineering Sciences, Huazhong University of Science and Technology, Wuhan, China; ^11^Tomsk State University, Laboratory of Biophotonics, Tomsk, Russian Federation; ^12^Institute of Precision Mechanics and Control of RAS, Laboratory of Laser Diagnostics of Technical and Living Systems, Saratov, Russian Federation; ^13^Humboldt University, Berlin, Germany; ^14^Potsdam Institute for Climate Impact Research, Potsdam, Germany

##### **Correspondence:** Oxana Semyachkina-Glushkovskaya - glushkovskaya@mail.ru

*Fluids Barriers CNS* 2019, **16(2):** A33

The high selectivity of the blood–brain barrier (BBB) is a hindrance to the entry of many drugs into the brain. It may at least partially explain why neurological disorders that continue to cause dramatic disabilities in a one billion people worldwide are so difficult to treat. Over the past four decades some progress has been made in overcoming the presence of the BBB. However, proposed approaches have been limited by a lack of specificity, safety concerns, inability to deliver sufficient drug concentration in the brain tissue, or difficulties in performing protocols. Therefore, the development of non-invasive approaches for brain drug delivery, which can be done in daily clinical practice, remains of critical importance in the treatment of brain disorders. Here we propose a quite novel and safe method for initiating BBB opening to low/high weight molecules and nanocarriers. This method is based on 2 h of exposure to loud music or otheraudible sound being produced by conventional loudspeakers or potential other audio devises (MP3 players etc.) through headphones. We discuss in details the systemic and molecular mechanisms underlying music/sound-BBB opening in ex vivo and in vivo experiments as well as in in vitro BBB models. We demonstrate in experiments on mice that sound-induced opening of the BBB is an easily performed method for safe delivery of drugs to the brain with high prospects of being widely applied in daily clinical practice for alternative therapy of Alzheimer and Parkinson diseases, depression, cognitive decline, and a post-surgical treatment of glioma. This reported music/sound-opening of the BBB in the intact brain warrants a revision of our traditional knowledge about the BBB phenomenon and opens a novel vision on the non-invasive strategies in brain drug delivery. It also may offer some insight into the etiology of brain disorders that follow inadvertent of deliberate exposure to very loud sounds, i.e. battle or rock concerts.

**Grant support:** This work was supported by the Grant of Russian Science Foundation 17-15-01263.

